# Sprayed biodegradable liquid film improved the freezing tolerance of cv. Cabernet Sauvignon by up-regulating soluble protein and carbohydrate levels and alleviating oxidative damage

**DOI:** 10.3389/fpls.2022.1021483

**Published:** 2022-11-01

**Authors:** Xing Han, Fei Yao, Ting-ting Xue, Zhi-lei Wang, Ying Wang, Xiao Cao, Miao Hui, Dong Wu, Yi-han Li, Hua Wang, Hua Li

**Affiliations:** ^1^ College of Enology, Northwest A & F University, Yangling, China; ^2^ School of Wine, Ningxia University, Yinchuan, China; ^3^ China Wine Industry Technology Institute, Yinchuan, China; ^4^ Shaanxi Engineering Research Center for Viti-Viniculture, Yangling, China; ^5^ Engineering Research Center for Viti-Viniculture, National Forestry and Grassland Administration, Yangling, China

**Keywords:** biodegradable liquid film (BLF), low temperature stress, freezing tolerance, mortality, antioxidant system, osmoregulation, C-repeat binding factor

## Abstract

Most cultivars of *Vitis vinifera* L. are very sensitive to cold. As an exogenous protectant, Biodegradable Liquid Film (BLF) is considered to protect winegrapes from low temperatures and dry winds for safe overwintering. This study aimed to reveal the physiological and biochemical mechanisms of BLF regulating the freezing tolerance of wine grapes. Groups of ten-year-old vines (Cabernet Sauvignon) were sprayed with BLF in November 2020 and 2021, or left untreated as a control treatment, and field plant mortality after overwintering were investigated. Branch samples were collected monthly for determination of biochemical indicators. Dormant two-year-old cuttings (Cabernet Sauvignon) were also used for the determination of relative expression levels of key genes. The results showed that the application of BLF reduced the branch semi-lethal temperature in January and February samples compared with control, and reduced the mortality of above-ground parts, branches and buds. The physiological status of shoots was greatly affected by the climatic conditions of the year, but BLF treatment increased the levels of soluble protein and soluble sugar, and also decreased the content of superoxide anion and malondialdehyde at most sampling times. Correlation analysis showed that the differences in freezing tolerance between BLF and no treated overwintering(CK) vines were mainly related to peroxidase activity, soluble sugar, reducing sugar and starch content. Low temperature stress activated the over expression of *ICE1*, *CBF1*, and *CBF3*, especially for 12h. BLF treatment significantly increased the expression levels of *CBF1* and *CBF3* under low temperature stress. Overall, these results demonstrate that BLF treatment protects vines from freezing damage by upregulating osmo-regulatory substances and alleviating oxidative damage.

## Introduction

Low temperature stress is one of the most important abiotic stresses, adversely affecting plant growth and development, restricting the geographic distribution of plant species, and reducing global crop yields (Lv et al., 2010; Kaya, 2020a; Kaya and Kose, 2020; Kaya, 2020b). Plants have evolved sophisticated mechanisms to withstand cold stress, such as cold acclimation, a process by which plants acquire increased freezing tolerance upon prior exposure to nonlethal low temperature (Guy, 1990; Thomashow, 1999; Chinnusamy et al., 2007). Under low temperature stress, the cell membrane system induces and transmits signals to the cell, prompting physiological and biochemical changes in the cell environment. The first is the leakage of electrolytes caused by the phase transition of the cell membrane (Lyons, 1973). In response to osmotic stress, soluble proteins, amino acids, soluble sugars and other substances are mass synthesized in cells (Bhowmik et al., 2006; Soloklui et al., 2012; Abeynayake et al., 2015). In addition, a large number of reactive oxygen species and free radicals will accumulate in cells after low temperature stress, as signal molecules, induce plants to respond to stress and generate oxidative stress, and malondialdehyde as a product of membrane lipid peroxidation is often used to evaluate this a process (Rende et al., 2018). In the process of cellular antioxidant, enzymatic systems including superoxide dismutase (SOD), peroxidase (POD), catalase (CAT) play an important role (Lee and Chen, 1992; Hasanuzzaman et al., 2020), ascorbic acid (ASA) and glutathione (GSH) also help resist plant stress by acting as a circulating peroxide ion scavenging system (Anjum et al., 2012; Pei, 2012).

Among the numerous low temperature-responsive signaling pathways, the CBF-COR signaling pathway is the most complete one described. C-repeat binding factor/dehydration-responsive element-binding protein 1 (CBF/DREB 1) is induced by low temperature and play a key role in plant low temperature acclimation (Stockinger et al., 1997). CBFs protein directly binds to CORs promoter and induces its expression, thereforeimproving the low temperature tolerance of plants (Stockinger et al., 1997; Liu et al., 1998; Siddiqua and Nassuth, 2011). CORs mainly encode osmotic regulator synthases and cryoprotective proteins, including a series of functional genes such as COR, LTI and KIN (Yamaguchi-Shinozaki and Shinozaki, 1994; Shi et al., 2018). Inducer of CBF expression 1 (ICE1) is a MYC-like bHLH transcription factor that encodes a protein that activates the expression of CBFs at low temperature by binding to the MYC site (CANNTG) in the CBF1-3 promoter (Chinnusamy et al., 2003; Kim et al., 2015).

As a perennial plant, fruit trees are more at risk of freezing damage than other crops (Snyder et al., 2005; Kaya et al., 2020). As one of the earliest and largest fruit trees planted in the world, most commercial grape varieties are sensitive to cold, and to survive the winter safely in many cool continental climates, grapevines must be buried under the soil (Zhang et al., 2012). This method is commonly used in cold climates like Eastern Europe, Northern China, Minnesota, and Quebec (Khanizadeh et al., 2005). Soil-burial increases labor intensity and costs, can cause cane damage and disease, restricts mechanized production, and can damage the ecological environment when there is no cover crop between rows (Pierquet et al., 1977; Jolivet and Dubois, 2000; Wang, 2015; Han et al., 2021). Therefore, studies of the feasibility of other winter protective measures are needed to support the development of the wine industry in such regions.

Biodegradable liquid film (BLF) was used in this experiment is a humic acid film, which was originally developed as an environmentally friendly soil structure conditioner. BLF can improve the soil structure, regulate the physical and chemical properties of the soil, facilitate the growth and development of crops, promote a good growth and reproduction environment for microorganisms, promote the transformation and accumulation of soil organic matter, and enhance soil fertility (Wang et al., 2010; Lan et al., 2013). The humic acid component in BLF can enhance the ability of plants to resist stress, and has obvious cold-resistant and growth-promoting effects (Lu, 2006). The use of plastic mulch results in the accumulation of large amounts of polystyrene nanoplastics in agricultural soils, affecting plant growth and stress resistance. Recent studies have shown that polymethylmethacrylate nanoplastics can penetrate cell walls and accumulate in plant cells, limiting photosynthesis in barley, resulting in decreased activities of carbohydrate metabolism-related enzymes and antioxidant enzymes, reducing its low temperature tolerance (Wang et al., 2022). The unique benefit is that the application of BLF is environmentally friendly and it can be naturally degraded under the action of sunlight and soil microorganisms, therefore its use does not damage the ecological environment (Wang et al., 2007a; Xue, 2019a). Recently, Xue et al., (2019b) found that BLF can be used as an exogenous antifreeze for plants. After being sprayed on the surface of grape plants, it solidifies and forms a film, which improves the survival rate of multiple varieties in the winter without soil-burial (Xue et al., 2019b).

In this study, BLF was used as an exogenous protectant to protect grapevines from freezing damage during overwintering. The protective effect of BLF on vines in the field trial was evaluated by investigating mortality after overwintering and measuring electrolyte leakage. The levels of biochemical indexes and the relative expression of cold resistance-related genes were also determined to explore the mechanism of BLF. These results will verify the effect of BLF on vines under low temperature stress, and provide a theoretical basis for overwintering strategies in cold regions.

## Materials and methods

### Material, vineyard site and treatments

This field study was conducted in 2020 and 2021, on 10-year-old ‘Vitis vinifera L. cv. Cabernet Sauvignon’ grapevines grown in a commercial vineyard in Xia County, Shanxi Provience (lat. 35°24′N, long. 111°17′E, alt. 433 m), China. Xia County is located in a continental semi-humid monsoon climate zone, with an average annual temperature of 12.8°C, a frost-free period of about 205 days, and an annual sunshine duration of 2293.4 h However, the winter temperatures may plunge to −15°C or even lower, causing serious damage to grape dormant buds

Grapevines were planted with a spacing of 1.0 m between grapevines and 2.7 m between rows, and were trained using a crawled cordon training system. The vines used in the experiment were not pruned after the fall of leaves in autumn, but were instead pruned one week before the bleeding period in spring, leaving three buds on each shoot. The vines were divided into two treatment groups: no treated overwintering (CK) and BLF sprayed in November (BLF). A completely randomized design was adopted, with three replications, with 24 vigorous vines per experimental unit. The treated vines were from two neighboring plots, allowing direct comparison of differences among treatments in the same soil and climate conditions. One-year-shoots samples were collected on December 20, 2020, January 15, February 7, March 3, December 23, 2021 and January 17, February 13, March 9, 2022. For both CK and BLF treatments, 20 one-year-shootswere collected in each of 3 experimental repeat units. The samples collected in the field were divided into two groups, one group was used for the determination of moisture content and electrolyte leakage immediately after being brought back to the laboratory, and the other group was stored in a -80 °C refrigerator for the determination of other biochemical indicators.

Two-year-old cuttings of Cabernet Sauvignon cuttings were also used for the experiments. Grape seedlings were cultivated in substrates composed of garden soil, perlite and humus (2:1:1, v/v/v) and 16 h light in a greenhouse at 24/18°C (day/night)/8hdark photoperiod, the photosynthetic photon flux density is 160 μmol m^-2^·s^-1^. In October 2021, the cuttings were transferred to the field for cultivation until natural dormancy. The cuttings were divided into two groups, which were treated with no treatment (CK) and sprayed with BLF (BLF) in November. Electrolyte leakage from annual dormant shoots of cutting from both treatments was determined. The cutting of the two treatments were divided into 4 groups, and each group was placed in a high and low temperature alternating test chamber (Shanghai Lanhao Instrument Equipment Co., Ltd., China, YSGJS-408) with low temperature stress of -10 °C for 3h, 6h, 12h, and 24h respectively. After the low temperature treatment, the one-year-shootsof the cuttings were quickly cut off, frozen in liquid nitrogen, and stored in a -80 °C refrigerator for gene expression analysis. A completely randomized trial was used, with three biological replicates for each experimental group, with 24 grapevine plants per experimental unit.

The biodegradable liquid film used in the experiment is a commercial product that purchased from Shaanxi Yang Ling Mingrui Technology Company. It’s brown and creamy and forms a thin, brown, multi-molecular chemical protection film that wraps and encloses the one-year-shootssurface. The BLF protects the one-year-shootsin winter and gradually degrades in spring (Xue et al., 2019b). The amount of BLF sprayed per hectare was 150 kg, which was diluted at the ratio of BLF: water = 1:3 (v/v) when used, and a gasoline sprayer was used for spraying. In this study, the film was applied to the one-year-shoots to be retained in the following year, trunks, and furrow soils.

### Mortality of vine, one-year-shoot and bud

**Figure 1:**
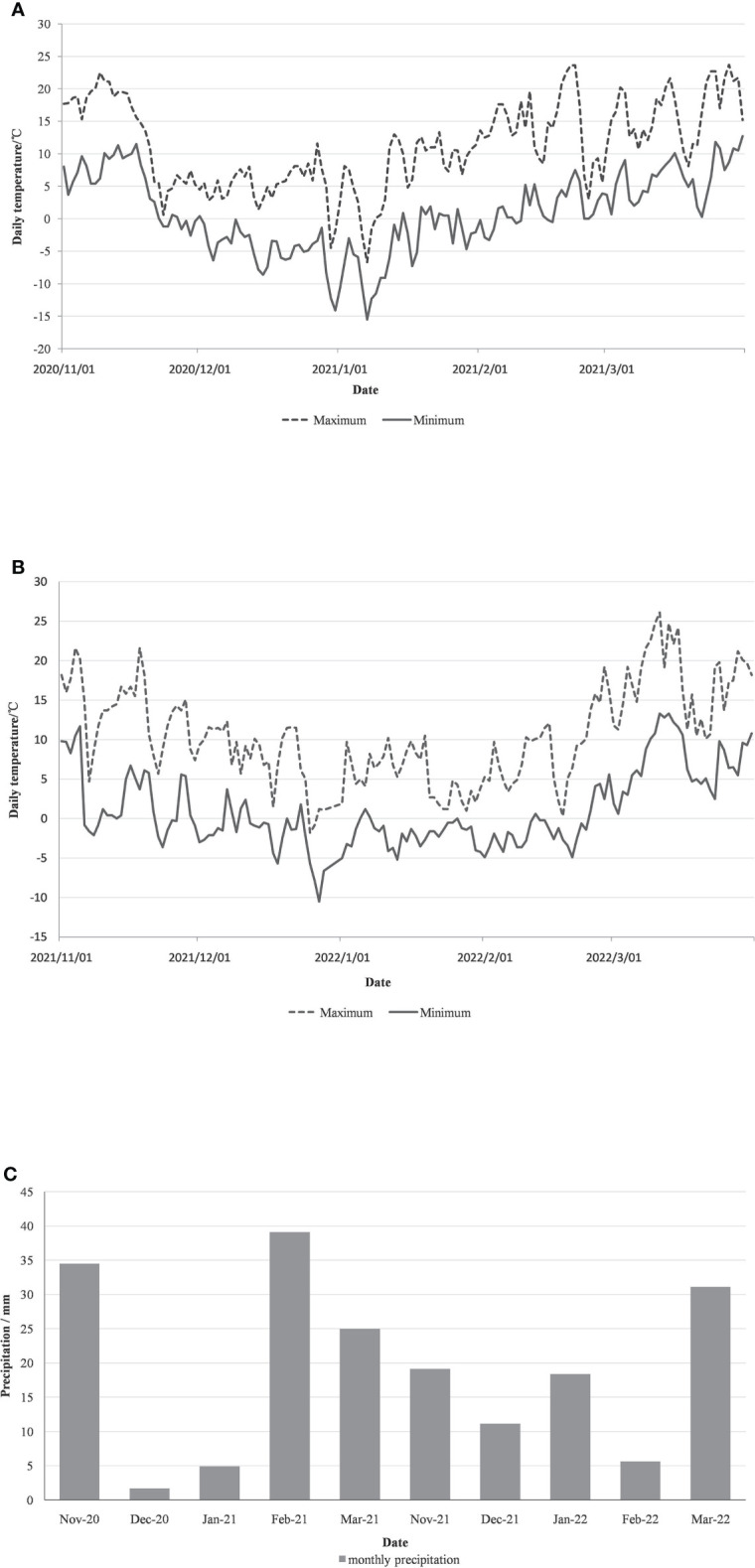
Daily maximum temperature, minimum temperature **(A, B)** and monthly precipitation **(C)** during the overwintering period (December to March) in 2020 and 2021 in Xia County.


The daily minimum and maximum temperature and monthly precipitation data were collected for the experiment site from December to March (**Figure 1**). And the mortality rates of whole vine, above-ground parts, one-year-shoots, and buds of vines under the two overwintering treatments were determined in April 2021 and 2022. During the investigation, the buds did not germinate, and the branches without sap flow in the tissue after cutting were counted as dead. To measure branch mortality, the germination of all one-year-shoots was determined for 10 randomly selected germinating trees. Bud mortality was measured by determining the germination of 10 randomly selected germinating one-year-shoots The mortality rate of each part is calculated according to Equation1-4:

(1) $$\text{Death rate of whole vine }\left(\%\right)=\frac{\text{the number of vines with dead above ground parts and no sprouting roots}}{\text{ total number of vines}}\times 100\text{\% }$$

(2) $$\text{Above ground part mortality rate }\left(\%\right)=\frac{\text{the number of vines whose roots are sprouting but the above ground part is dead}}{\text{total number of vines}}\times 100\%$$

(3) $$\text{Average one–year–shoot mortality rate }\left(\%\right)=\frac{\text{the number of dead branches on }10\text{ trees}}{\text{total number of branches on }10\text{ trees}}\times 100\text{\% }$$

(4) $$\text{Average bud mortality rate }\left(\%\right)=\frac{\text{the number of dead buds on }10\text{ branches}}{\text{total number of buds on }10\text{ branches}}\times 100\%$$

### Freezing procedure

Low temperature treatment was carried out using a high and low temperature alternating test chamber (YSGJS-408). Treatment temperatures for determination of electrolyte leakage were 4, -6, -10, -14, -18, -22 and -26°C, with a cooling rate of 4°C per hour. Samples were maintained at the set temperature for 12h The temperature was then restored to 4°C at 4°C per hour. The samples were maintained at 4°C to recover for 12hbefore removal from the chamber and recovery to room temperature to measure electrolyte leakage (Wang et al., 2007b; Hashempour et al., 2014). The procedure of low temperature stress for cutting was to drop from room temperature to -10 °C at a rate of 4 °C/h, and keep at -10 degrees for 3h, 6h, 12h, and 24h, and then collect the samples.

### Determination of electrolyte leakage

The relative electrical conductivity was determined and the lethal temperature (LT) at which 50% of the total electrolyte leakage occurred in branch tissues (EL-LT50) was calculated according to the method of Wang et al. (2007b) with slight modifications. To avoid the influence of BLF materials, the one-year-shootsfor both treatments were cut off the epidermis, and cut into 0.3-0.5 cm thick slices. Samples (weighing 2.0 g) of the branches were cut and placed into a 25 mL test tube with a stopper. Next, 20 mL of distilled water was added, the tube was stoppered and then shaken in a shaker for 12 h before the conductivity (μS•cm^-1^) was measured using a conductivity meter (DDS-307A). The test tubes were then boiled for 40 min, allowed to stand for 2 h, and then the conductivity value was re-measured. The relative conductivity was measured according to Equation 5:


(5) $$\text{Relative electrical conductivity }\left(\%\right)=\frac{\text{conductivity value before boiling}}{\text{conductivity value after boiling}}\times 100\%$$

The improved logistic equation was used to fit the conductivity values (Equation 6):


(6) $$Y=\frac{\text{K}}{1+{\text{ae}}^{-\text{bx}}}$$

The second derivative of the equation was obtained and set equal to zero, where K=100. The inflection point of the equation curve X=lna/b, and X is the EL-LT50 of the branch.

### Determination of branch water content

The total water content was determined by the drying method. Collected branches were cut into 3 cm sections, accurately weighed, and killed by baking at 105°C for 0.5 h. The samples were then baked at 80°C for 16 h to achieve constant weight and the tissue water content was determined.

Free water content was measured according to the method of Chen et al. (2014). Collected branches were cut into slices of about 1 mm. Three 0.5g samples were transferred into three weighing bottles, accurately weighed, and 5mL of 65% sucrose solution was added before accurate weighing again to calculate the weight of the sugar solution. The sucrose-containing bottles were incubated in the dark for 4-6h, with occasional shaking before measurement of the sugar concentration using an pocket refractometer (PAL-1) to determine the concentration of sugar solution. The free water content was calculated according to the sucrose concentration before and after soaking, and the bound water content was the difference between the total water content and the free water content.

### Determination of oxidative stress indices

Superoxide anion (O^2-^) production was estimated as described by Elstner and Heupel (Elstner and Heupel, 1976).

Malondialdehyde (MDA) content was determined by thiobarbituric acid-reactive substances method (Hodges et al., 1999). Branch sample (2.0 g) was homogenized in 15 mL 0.1% TCA and then centrifuged at 5,000 rpm for 10 min. Next, 5 ml of 5% TCA containing 0.5% TBA was added to 1 mL of the supernatant, followed by incubation in boiling water for 10 min and then transfer to ice water to stop the reaction. MDA absorption was measured spectrophotometrically at 450, 532, and 600 nm.

### Determination of anti-oxidative enzymes activities

Branch samples (1.25 g) from the two treatment groups were ground in a chilled mortar with 1% (w/v) polyvinylpolypyrrolidone, homogenized with 30 mL of 50 mM potassium phosphate buffer (pH 7.8), and then centrifuged at 10,000 rpm for 15 min. The supernatant after centrifugation was used for the determination of antioxidant enzyme activities. The supernatant after centrifugation was assayed for antioxidant enzyme activity. Superoxide dismutase (SOD; EC 1.15.1.1) activity was estimated by NBT and was expressed as U·g^-1^FW·h^-1^ (Giannopolitis and Ries, 1977). Catalase (CAT; EC 1.11.1.6) activity was evaluated according to the decomposition rate of hydrogen peroxide and was expressed as U·g^-1^FW·min^-1^ (Aebi, 1984). Peroxidase (POD; EC 1.11.1.7) activity was measured by guaiacol and was expressed as μg·g^-1^FW·h^-1^ (Rao et al., 1996).

### Determination of osmoregulation substances

The extraction of soluble protein was according to the same procedure used to extract the antioxidant enzymes. The resulting supernatants were assayed for soluble protein content by Coomassie brilliant blue method, using bovine serum albumin as a standard (Bradford, 1976) and detection of protein at 525 nm.

Proline content was measured based on the method of (Bates et al., 1973). A branch sample (0.5 g) was placed into 5 mL of 3% aqueous sulfosalicylic acid, incubated in a boiling water bath for 30 min, cooled to room temperature, and centrifuged at 8,000 rpm for 5 min. Next, 1.0 mL of the supernatant extract was mixed with 2 mL ninhydrin and 2 mL acetic acid, maintained for 30 min in a boiling water bath, and then cooled to room temperature. Next, 5 mL of toluene was added, and the tube was turned upside down and mixed well before being placed in the dark for 3 h. The colored product with toluene was detected at 520 nm.

### Determination of carbohydrate

Collected branch samples were dried to a constant weight and then crushed for the determination of sugar content. Reducing sugar content was measured using the 3,5-dinitrosalicylic acid method to determine the absorbance at 520 nm and calculated based on the standard curve of glucose (Gao, 2006). The soluble sugar content was determined by anthrone colorimetry at 620 nm and calculated according to the standard curve of glucose (Yemm and Willis, 1954). The reducing sugar extract was hydrolyzed with 6 mol/L hydrochloric acid and then neutralized with sodium hydroxide for the determination of sucrose content using 3,5-dinitrosalicylic acid method (Gao, 2006). The residue left after the extraction of reducing sugar was hydrolyzed with hydrochloric acid and then neutralized with sodium hydroxide for the determination of starch content. The starch content was determined using the 3,5-dinitrosalicylic acid method, as was conductedto measure reducing sugar content (Gao, 2006).

### Transcriptional analysis of genes by real-time quantitative polymerase chain reaction

Branch tissue (100 mg), previously ground in liquid N^2^, was used for total RNA extraction. Total RNA was extracted from each sample with Quick RNA Isolation Kit (Huayueyang, Beijing, China), following the manufacturer’s instructions. The integrity and purity of the RNA were detected by the 2100 bioanalyzer and photometer (Agilent,USA). Afterward, cDNA was synthesised from 1 μg of total RNA using HiScript III 1st Strand cDNA Synthesis Kit (Vazyme, Nanjing, China). Real-time PCR analysis was performed with ChamQ Universal SYBR qPCR Master Mix (Vazyme, Nanjing, China) using 2 μL cDNA in a final reaction volume of 20 μL per well. The 20μL reaction system includes: 2×ChamQ Universal SYBR qPCR Masher Mix (10.0μL), Primer1 (10μM, 0.4μL), Primer2 (10μM, 0.4μL), cDNA (2μL), ddH2O (7.2μL). The qPCR was conducted on a qTOWER3G (Analytik Jena, Jena, Germany). The reaction procedure was ① 95°C for 3 min; ② 95°C for 10 s, then 60°C for 30 s, and step ② for 40 cycles; ③ 95°C for 15s, 60°C for 60s, and 95°C for 15s. Genes selected for further analysis included *VvICE1* (inducer of *CBF* expression 1), *VvCBF1* (c-repeat binding factor 1), *VvCBF3* (c-repeat binding factor 3). *Actin* was used as the internal control. Primer sequences designed by Primer 5 are shown in **Table 1**. Specific gene amplification was confirmed by melting curve, agarose gel and sequencing analysis. The expression values were normalised by the average of the expression of the reference genes and calculated using the 2^-ΔΔCt^ method (Livak and Schmittgen, 2001).

**Table 1:** Sequences for primers used in qRT-PCR.

Gene	Primer sequencece (5’- 3’)
*Actin*	F: 5’-GTGCCTGCCATGTATGTTGCC-3’ R: 5’-GCAAGGTCAAGACGAAGGATA-3’
*VvICE1* inducer of CBF expression 1	F:5’- GCCAAGGGTTGAAGTGA-3’ R:5’- CCGCCTGTTGAACGTCTAG-3’
*VvCBF1* c-repeat binding factor 1	F:5’-GGGGCGAAGGTCGTGTT-3’ R:5’- GTCCCATCGTTTCCATTTT-3’
*VvCBF3* c-repeat binding factor 3	F:5’- GCATAAGCGGAAAGCAG-3’ R:5’- CCGCACTTCGCATACCC-3’

### Statistical analysis

All data were evaluated by one-way ANOVA. Multiple comparisons tests were taken only between treatments with Duncan’s multiple comparison test, where differences were considered significant at P ≤ 0.05. And graphs were performed by GraphPad prism software 6.0 (Graph Pad Software, San Diego, CA, USA).

The Pearson’s coefficient of correlation analysis was determined between the levels of each indicator, correlation significance was defined at the 0.05 level. The graph was created by the genescloud tools, a free online platform for data analysis (https://www.genescloud.cn).

## Results

### Climatic conditions and mortality in the field trial

The field experiment was conducted in two wintering periods of 2020-2021 and 2021-2022. The daily extreme temperature and monthly precipitation of the experiment site were provided by the Great Winery. The minimum temperature during the wintering season in 2020 is -15.5°C, and the precipitation is mainly in November, February, and March, and there is almost no precipitation in December. The minimum temperature during the wintering period in 2021 is only -10.5°C, with precipitation in every month, and the climate is milder than that in 2020.

The survival of vines under the two treatments was investigated in 2021 and 2022, with measurement of whole tree, above-ground, branch, and bud mortality. During the investigation, vines for both treatment groups were in the germination period. There was no whole tree mortality in either treatment, but higher mortality of above-ground parts for the untreated plants for two years. The mortality of branch and bud of vinesprotected by BLF is obviously lower in 2020 (**Table 2**). The difference in bud mortality between the two treatments in winter 2021 was not large, probably because the minimum temperature in winter was higher, which did not cause serious freezing damage (**Figure 1**).

**Table 2:** The effect of spraying BLF on vine, one-year-shoot, and bud mortality.

Treatment	Whole vine mortality	Above-ground part mortality	One-year-shoot mortality	Bud mortality
CK-2020	0	10.00%	6.38%	25.93%
BLF-2020	0	1.75%	5.45%	10.71%
CK-2021	0	4.00%	4.00%	9.52%
BLF-2021	0	2.00%	2.00%	8.70%

### Electrolyte leakage of branches in the field trial

The EL-LT50 value was calculated based on the relative electrical conductivities. During the overwintering period, the EL-LT50 values of the branches for both treatments first decreased and then increased, reaching the minimum value in January, corresponding to the maximum freezing tolerance (**Table 3**). The EL-LT50 of BLF-treated branches in January was significantly lower than that of CK both 2020 and 2021. There was no significant difference between the two treatments of EL-LT50 in March, which may be related to the natural degradation of the BLF material and the release of dormancy.

**Table 3:** Effect of spraying BLF on the EL-LT50 value (°C) of one-year-shoots (estimated by relative electrical conductivities).

Treatment	December	January	February	March
CK-2020	-12.72a	-14.27b	-14.05a	-13.35a
BLF-2020	-12.24a	-16.92a	-14.76a	-12.38a
CK-2021	-13.93a	-16.8b	-11.85b	-9.82a
BLF-2021	-14.68a	-17.87a	-13.09a	-10.11a

Mean values for each parameter (EL-LT50) followed by the same lower-case letters in each column are not significantly different at P ≤ 0.05 by Duncan’s multiple range test.

### Water content of branches in the field trial

**Figure 2:**
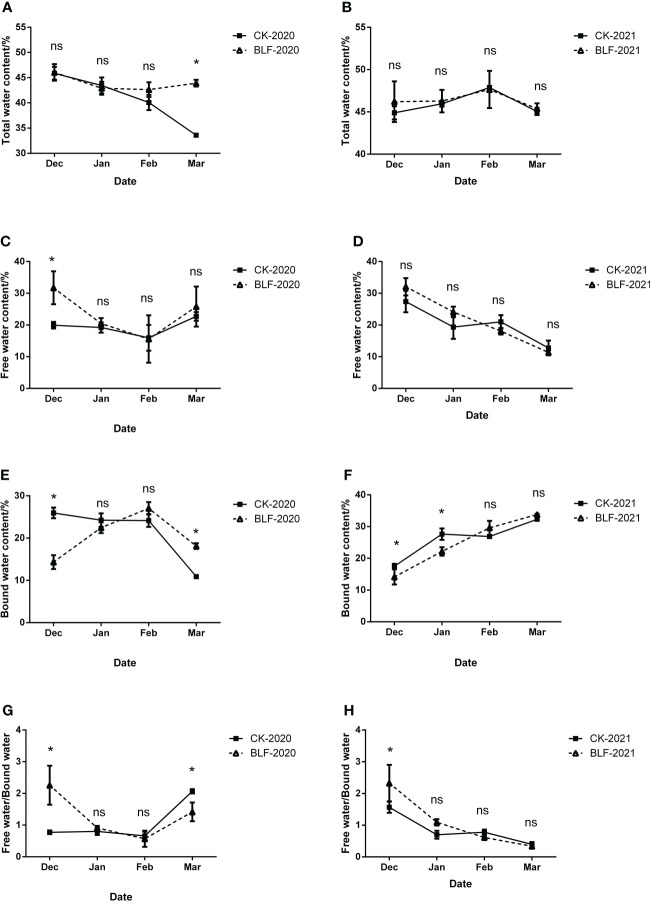
The effect of spraying BLF on the content of total water **(A, B)**, free water **(C, D)**, bound water **(E, F)**, and the ratio of free water to bound water **(G, H)** on 2020 and 2021. “*” means that the average value of each parameter is significant between the two treatments (p<0.05), and ns means no significance by Duncan’s multiple range test.

The water content composition of the branches was measured during the overwintering period and the results are shown in **Figure 2**. Under the influence of the continental monsoon climate, the dry and cold wind caused water loss of vines and the total water content gradually decreased. However, there was less water loss of the BLF-treated plants than that of the control, and this trend became more obvious over time between CK-2020 and BLF-2020. There was no significant difference in the total water content of the two treatments in the early overwintering period, but there was higher total water content in the branches sprayed with BLF in the late overwintering period, with a significant difference on March 3, 2021 (**Figure 2A**).

During the overwintering period, the moisture composition of the vine branches have significantly difference between two years. The content of free water decreased in December, January, and February, but increased in March, while the bound water content exhibited the opposite trend during 2020 winter period. Except for December 20, 2020, there were no significant differences in the free water content, bound water content, and free water/bound water of the two treatments (**Figure 2**). There was no significant difference in the water composition of the two treatments in the 2021 overwintering period except for the bound water content in December 2021 and January 2022 (**Figure 2**).

### Oxidative damage of branches in the field trial

**Figure 3:**
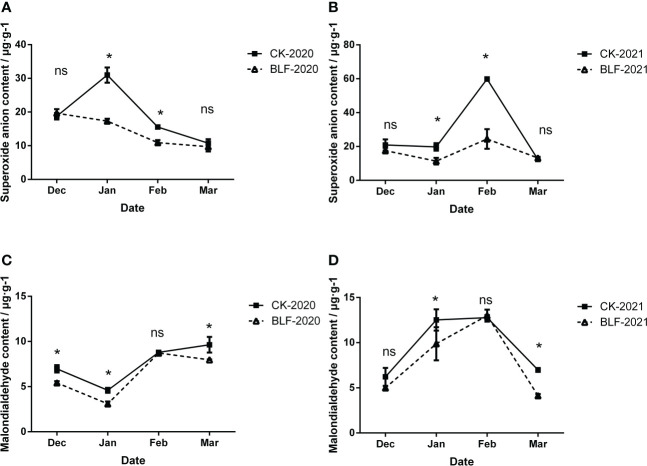
Effect of spraying BLF on superoxide anion **(A, B)** and malondialdehyde **(C, D)** on 2020 and 2021. “*” means that the average value of each parameter is significant between the two treatments (p<0.05), and ns means no significance by Duncan’s multiple range test.

Low temperature stress increased the degree of membrane lipid peroxidation in cells, which was reflected in the increase of superoxide anion and malondialdehyde content during overwintering. There was no significant difference between the two treatments in December and March, but in January and February, the superoxide anion content of the control plants was significantly higher than the plants that received the BLF treatment both of two years (**Figures 3A, B**). Compared with that of CK, the maximum decrease of superoxide anion content in BLF treatment at two years was 44.12% (**Figure 3A**) and 59.16% (**Figure 3B**), respectively. As shown in **Figure 3C**, except for February, the MDA content was always higher for the CK plants than for the plants that received the BLF treatment during the wintering period in 2020. During the overwintering period in 2021, the MDA content of BLF treatment was also significantly lower than that of CK in January and March (**Figure 3D**). During the wintering period of 2020, the MDA content basically showed an upward trend, while in 2021 it first increased and then decreased, which may be related to the temperature difference between the two years. The extreme minimum temperature of the wintering period in 2020 reached -15.5°C, and the oxidative damage to the cell membrane has been accumulating. The minimum temperature of the wintering period in 2021 is only -10.5°C, and the cell membrane gradually recovers its activity in the late winter.

### Antioxidant enzyme activity and antioxidant content of branches in the field trial

**Figure 4:**
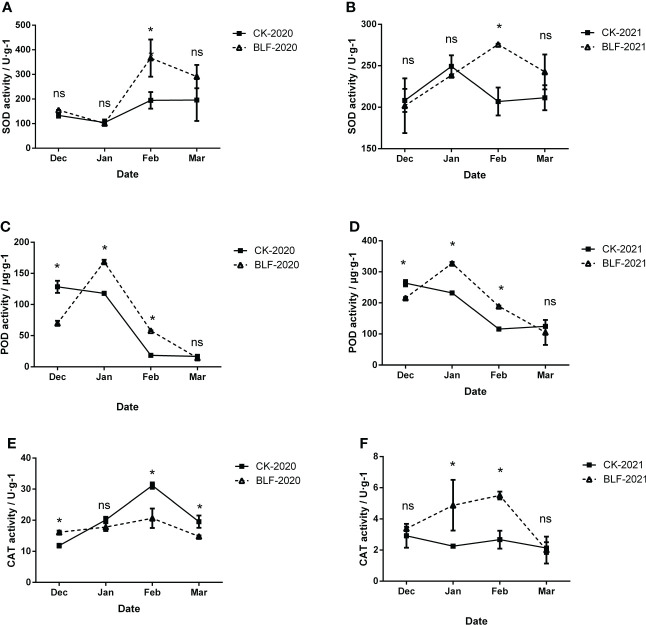
Effect of spraying BLF on superoxide dismutase **(A, B)**, peroxidase **(C, D)**, and catalase **(E, F)** activities on 2020 and 2021. “*” means that the average value of each parameter is significant between the two treatments (p<0.05), and ns means no significance by Duncan’s multiple range test.

To evaluate the effect of BLF treatment, the activities of superoxide dismutase (SOD), peroxidase (POD), and catalase (CAT) were measured, as shown in **Figure 4**. In both years, the activity of SOD reached the highest in February, and the SOD activity of BLF treatment was significantly higher than that of CK, and there was no significant difference at other times (**Figures 4A, B**). The trend in POD activity was very consistent between the two years, regardless of CK or BLF treatment (**Figures 4C, D**). The POD activity of BLF treatment first increased and then decreased in both wintering periods, and reached the highest in January, while the CK treatment has been in a downward trend. The POD activity was significantly higher in the BLF treatment than for CK in January and February. The trend of CAT activity in two years was basically opposite (**Figures 4E, F**,).

**Figure 5:**
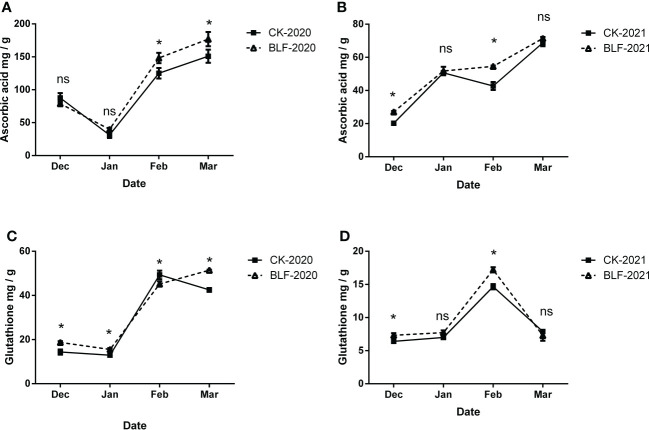
Effect of spraying BLF on the content of ascorbic acid **(A, B)** and glutathione **(C, D)** on 2020 and 2021. “*” means that the average value of each parameter is significant between the two treatments (p<0.05), and ns means no significance by Duncan’s multiple range test.

Antioxidants are key to the non-enzymatic reaction system, and ascorbic acid (ASA) and reduced glutathione (GSH) were measured in this study (**Figure 5**). During overwintering period in 2020, there was no significant difference in ASA content for the two treatments in December and January, but BLF increased more than CK, with a significant difference in February and March (**Figure 5A**). The content of GSH also increased sharply in the late over-wintering period. The difference was that the BLF treatment continued to increase, while the CK treatment decreased slightly in March (**Figure 5B**). There was only a significant difference between the two treatments in February and March. In 2021, the changing trends of both ASA and GSH have changed, with ASA content increasing over time and reaching the highest level in March, while GSH content reaching the highest level in February and dropping sharply in March (**Figures 5C, D**). However, the contents of ASA and GSH of BLF treatment in December and February were significantly higher than those of CK.

### Osmoregulation substances of branches in the field trial

**Figure 6:**
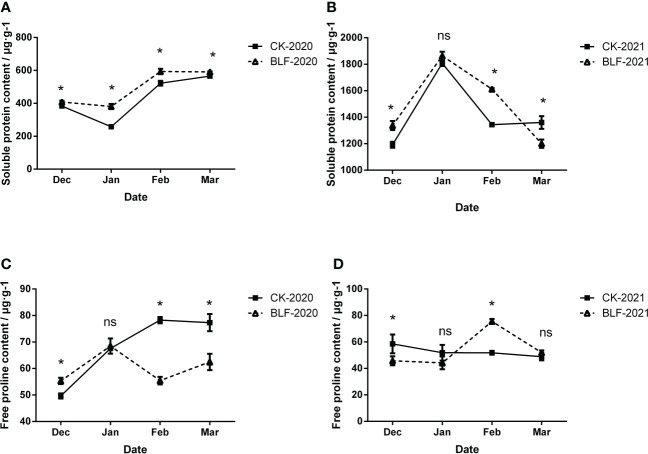
Effect of spraying BLF on the content of soluble protein **(A, B)** and free proline **(C, D)** on 2020 and 2021. “*” means that the average value of each parameter is significant between the two treatments (p<0.05), and ns means no significance by Duncan’s multiple range test.

Substances that allow osmotic adjustment are part of the stress response in plants, including free proline, soluble protein, and soluble sugar, etc. Measurements of the soluble protein and free proline content for the two treatments during the overwintering period are shown in **Figure 6**. BLF treatment up-regulated the soluble protein content of the branches in most cases, especially in 2020, the soluble protein content of BLF was significantly higher than that of CK throughout the wintering period (**Figure 6A**). However, the change trend of soluble protein content in the two years was different (**Figures 6A, B**). The free proline content increased first and then decreased for the two treatments in 2020 (**Figure 6C**). Initially, the proline content of BLF-treated plants was higher, but in February and March in the late overwintering period, the level was significantly higher for the CK-treated plants. During the overwintering period in 2021, the changes in the proline content of BLF treatment were similar to those in 2020, which also showed a trend of first increasing and then decreasing, while the proline content of CK was basically stable (**Figure 6D**).

### Carbohydrate content of branches in the field trial

**Figure 7:**
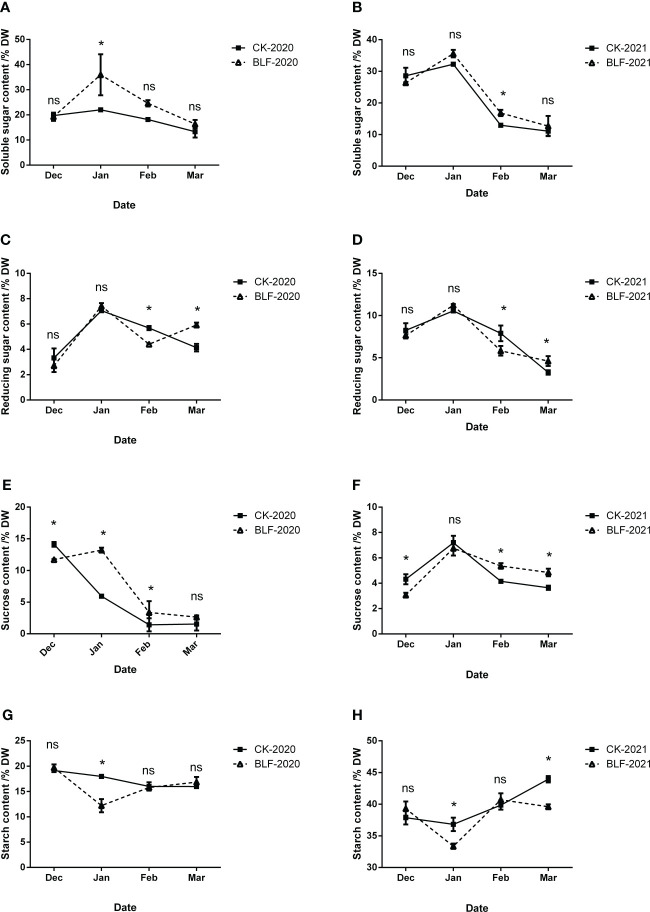
Effect of spraying BLF on the content of soluble sugar **(A, B)**, reducing sugar **(C, D)**, sucrose **(E, F)** and starch **(G, H)** on 2020 and 2021. “*” means that the average value of each parameter is significant between the two treatments (p<0.05), and ns means no significance by Duncan’s multiple range test.

The content of soluble sugar showed a trend of increasing first and then decreasing during the wintering period, with the highest value in January in both of two years (**Figures 7A, B**). The range of soluble sugar changes in BLF treatment was greater than that of CK, and the two were significantly different in January during 2020 wintering period, but not so much in 2021. In general, the change trend of reducing sugar content was similar to that of soluble sugar, with the maximum value in January (**Figures 7C, D**). There was no significant difference between the two treatments in December and January. Except for March, the reducing sugar content of BLF treatment was always lower than that of CK. During the wintering period, the sucrose content first decreased and then increased, reaching the minimum in February except for CK-2020 (**Figures 7E, F**). During the overwintering period in 2020, the sucrose content of BLF was always higher than that of CK except in December, and the difference was not significant in March. During the overwintering period in 2021, the sucrose content of BLF treatment was also significantly lower than that of CK in December, and significantly higher in February and March. Similar to the soluble sugar content, the change in starch content of BLF treatment was much greater than that of CK, with a significant difference between the two in January both of two years (**Figures 7G, H**). The starch content of BLF-2021 was also significantly lower than that of CK-2021 in March (**Figure 7H**).

### Correlation analysis between freezing tolerance and biochemical indicators

**Figure 8:**
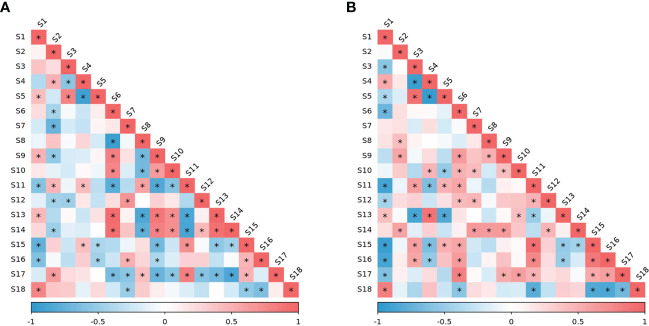
Pearson correlation values between the EL-LT50 (in °C) of vine one-year-shoots and other indicators under BLF and CK treatments in overwintering period of 2020 **(A)** and 2021 **(B)**. S_1_: EL-LT50; S_2_: total water; S_3_: free water; S_4_: bound water; S_5_: free water/bound water; S_6_: soluble protein; S_7_: free proline; S_8_: superoxide anion; S_9_: malondialdehyde; S_10_: superoxide dismutase; S_11_: peroxidase; S_12_: catalase; S_13_: ascorbic acid; S_14_: reduced glutathione; S_15_: soluble sugar; S_16_: reducing sugar; S_17_: sucrose; S_18_: starch. “*” means that the correlation between the two indicators is significant at the 0.05 level.

The person correlation coefficient between the level of each physiological and biochemical index and EL-LT50 was analyzed (**Figures 8A, B**). During wintering period in 2020, free water/bound water, MDA, ascorbic acid, and starch were significantly positively correlated with EL-LT50, indicating that the higher their level, the larger the value of EL-LT50. The value of EL-LT50 is a negative number, and the larger the value, the higher the semi-lethal temperature and the poor freezing tolerance. However, POD, soluble sugar and reducing sugar were all significantly negatively correlated with EL-LT50, indicating that the higher their content, the lower the value of EL-LT50, and the better the freezing tolerance. During wintering period in 2021, bound water, ascorbic acid, and starch were significantly positively correlated with EL-LT50, and more indicators were significantly negatively correlated with EL-LT50, including free water, free water/bound water, soluble protein, POD, CAT, soluble sugar, reducing sugar and sucrose. The correlation analysis results of the two years are quite different, especially the composition of moisture content, because it is affected by climatic conditions such as precipitation. Nevertheless, the activities of antioxidant enzymes, especially POD, were significantly negatively correlated with EL-LT50 in both years, indicating that it plays a key role in alleviating oxidative stress. The correlations of carbohydrates in the two years were very consistent. Soluble and reducing sugars have always been significantly negatively correlated with EL-LT50, sucrose also showed a significant negative correlation in 2021, and starch was always significantly positively correlated with EL-LT50. This indicated that the effect of BLF on plant freezing tolerance was mainly related to antioxidant enzyme activity and carbohydrate content.

### Relative expression levels of the genes under low temperature stress

**Figure 9:**
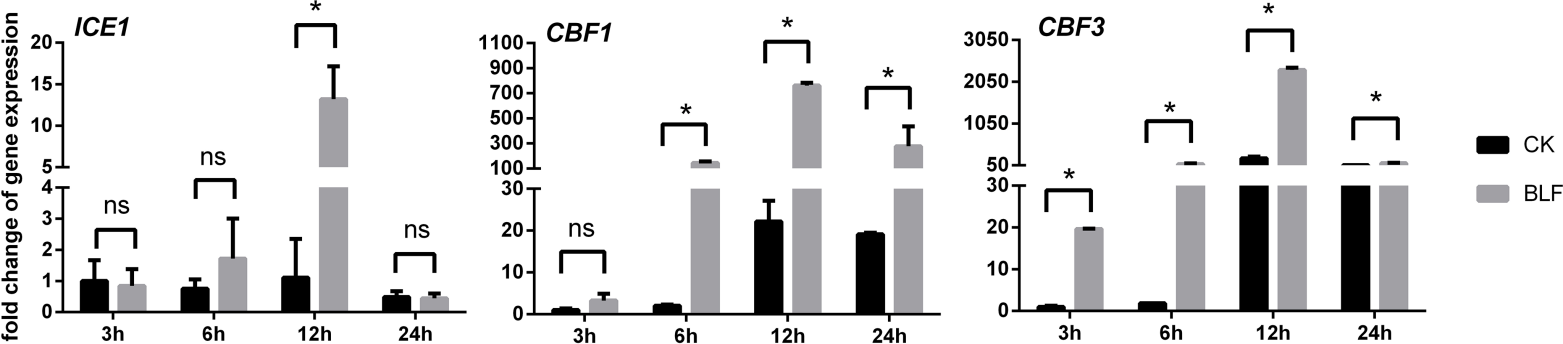
Effect of spraying BLF on the relative expression levels of genes associated with freezing tolerance. “*” means that the average value of each parameter is significant between the two treatments (p<0.05), and ns means no significance by Duncan’s multiple range test.

Due to the difference in growth vigor, age and cultivation environment, the freezing tolerance of cuttings and perennial vines in the field was very different. The electrolyte leakage of dormant cuttings branches was first measured in this study (not shown in the figure and table), the EL-LT50 of CK was -9.84°C, and that of BLF treatment was -11.70°C, so -10°C was selected as the low temperature stress temperature to simulate field conditions. Under freezing temperature stress, the expression levels of CBF1 and CBF3 were basically the same with the stress time. Both CK and BLF treatments reached the peak at 12h and decreased at 24h, which may be due to prolonged extreme stress leading to plant death, cell membrane rupture, and intracellular transcription and metabolism disorders (**Figure 9**). The effect of BLF on the expression levels of CBF1 and CBF3 was very significant, except for CBF1 at 3h. At 6h, 12h and 24h, the relative expression of CBF1 in BLF-treated branches was 71.88, 34.32, and 14.53 times that of CK, respectively. Similarly, at 3h, 6h, 12h and 24h, the relative expression of CBF3 in BLF-treated branches was 19.66, 43.91, 10.48, and 5.56 times that of CK, respectively. The relative expression of ICE1 in the branches of the two treatments only showed significant difference at 12h, and the BLF treatment was significantly higher than that of the CK.

## Discussion

Under low temperature stress, plants need to maintain cell membrane stability and biologically active protein structures to survive in unfavorable environments (Chen et al., 2018; Kaya et al., 2021a; Kaya et al., 2021b). Cold acclimation is an important process enabling many plants to cope with low temperature stress. Acclimation includes many physiological and biochemical changes in cells driven by shortened sunlight and reduced temperature. There is an interaction between low temperature exposure and short-day sunlight (Zhang et al., 2002), low temperature further increases the bud freezing tolerance of photoperiod-responsive genotypes and can also induce dormancy and domestication of non-photoperiod-responsive varieties (Meiering et al., 1980; Schnabel and Wample, 1987).

### BLF treatment reduced water loss and electrolyte leakage of overwintering vines

Exposure of plants to sub-zero temperatures can cause plant tissues to freeze (Puhakainen et al., 2004). Ice crystals first form in the extracellular space of plant cells, reducing the water potential of the apoplast solution, causing water to flow out of the cell, so freezing stress at the cellular level is usually accompanied by dehydration stress (Ritonga and Chen, 2020). Ice crystals can increase electrolyte leakage and membrane lipid phase changes (Vadim et al., 2014). In this study, controlled laboratory freezing tests were used to simulate the cooling process, during which there was electrolyte leakage and LT50 were calculated based on the relative electric conductivity (**Table 3**). With cold acclimation, the EL-LT50 showed a decreasing trend in the early overwintering period, demonstrating that the freezing tolerance of plants gradually increased, reaching maximum value in January. With the increase of temperature in February and March, deacclimation occurred and the freezing tolerance of plants was gradually lost, as seen in many previous studies (Yang et al., 2015; Haghi et al., 2019).

The hydrophobic film formed by BLF may also help to slow the formation of ice crystals. The general melting point of frozen plants is close to 0°C, but studies on various crops have shown changes in the freezing point of plants or supercooling capacity (Chen et al., 1995). Surface water contributes to the formation of early ice nuclei in plants (Fuller and Grice, 1998; Fuller and Wisniewski, 1998), and free water promotes the activity of ice nuclei in the leaf plane. Fuller et al. (2003) studied potatoes and showed that the hydrophobic granular film can prevent the formation of ice nuclei of leaves by delaying the ice penetration of frozen droplets on the leaf surface. A similar study of tomato plants reached the same conclusion (Wisniewski et al., 2002). Further tests of the effect of BLF on tissue icing are still needed.

Decreased water content is associated with increased bud hardiness in grapevine (Bourne and Moore, 1991; Salzman et al., 1996; Ershadi et al., 2016), but severe water loss may cause plant death (Chai and Wang, 1996). Under natural low temperature stress, the bound water content of plants increases and the free water content decreases, so the ratio of bound water to free water increases. This ratio is typically positively correlated with the cold resistance of plants (Chen et al., 2014). In China’s viticulture areas with continental monsoon climate, dry winds in winter are one of the main causes of death of grape plants (Liu et al., 2004; Li, 2015). Increased water retention capacity of grape branches can enable survival of winter. The use of BLF can significantly reduce the water loss rate of isolated branches by 3.12% (Xue et al., 2019b). In this study, the use of BLF significantly increased the total water content of the branches in the late overwintering period of 2020, and this effect may be related to the formation of a hydrophobic protective layer after BLF film formation. BLF limits gas exchange between the branches and the environment and slows the evaporation of water from the plants. There was lower mortality of the above-ground parts, branches, and buds of the plants treated with BLF compared to the control plants (**Table 2**), which may be related to changes in water retention.

### BLF treatment alleviated oxidative stress in overwintering vines by improving antioxidant systems

Significant damage can be caused by reactive oxygen species under low temperature stress. To cope with oxidative damage, plants have evolved a complete antioxidant defense system, including antioxidant enzymes (such as SOD, CAT, POD, APX, etc.) and antioxidants (such as AsA, GSH, sulfhydryl compounds, phytochelatins) (Liu et al., 2022). SOD converts O^2-^ into H_2_O_2_ and is considered the first line of defense against oxidative damage (Kaya and Köse, 2017). Then H_2_O_2_ can be converted to H_2_O by POD, CAT, and APX (Hasanuzzaman et al., 2020). In this study, BLF treatment reduced the O^2-^ and MDA content in branches during the overwintering period suggested that vines under low temperature stress treated with BLF maintained higher cell membrane integrity (**Figure 3**).

The scavenging enzymes are a key protein fraction in the acquisition of FT in plants (Lee and Chen, 1992). Plants under low temperature stress can alleviate reactive oxygen species damage by enhancing their antioxidant defense systems. The results of this study showed that the activity of antioxidant enzymes in grapevines tended to increase during cold acclimation, reaching a peak in January and February with cooler temperatures and decreasing during de-acclimation (**Figure 4**). This is consistent with many previous studies (Hashempour et al., 2014; Haghi et al., 2019) . ASA and GSH are also important components of the oxidative defense system, acting as antioxidant protection substances and maintaining the balance between ROS generation and scavenging by participating in the ASA-GSH cycling pathway (Anjum et al., 2012). In this study, the contents of ASA and GSH both increased significantly in the late overwintering period (**Figure 5**). Due to the variability of field climate and complex experimental conditions, the correlation analysis between oxidative damage and antioxidant system of branches during overwintering did not show a consistent law. However, the value of EL-LT50 was negatively correlated with the activities of POD and CAT in both years (**Figure 8**), indicating that the difference in FT between BLF and CK treatments may be mainly related to the scavenging of H_2_O_2_ by POD and CAT.

### BLF treatment increased the contents of Osmoregulation substances of overwintering vines

The changes that occur in plants under cold stress prepare cells and organs to tolerate apoplastic freezing and its dehydration consequences (Weiser, 1970; Uemura and Steponkus, 1994; Grossnickle and South, 2014). Soluble protein can improve the water holding capacity of cells, protect the plasma membrane structure, and increase the concentration of cell fluid to lower the freezing point and improve the cold resistance of plants (Li et al., 2011). The soluble protein content of BLF treatment in this study was consistently higher than that of CK except in March 2022 (**Figures 6A, B**).

Under cold stress, polysaccharides are hydrolyzed into soluble sugars, therefore increasing the osmotic potential of the cytoplasm and lowering the freezing temperature. Soluble sugars can increase the osmotic concentration of cells, reduce water potential, and increase water retention capacity, therefore lowering the freezing point to function as a protective agent for ice, with a protective effect on protoplasts, mitochondria, and sensitive coupling factors on membranes (Wisniewski et al., 2003; Ren et al., 2013; Ling et al., 2015). Ling et al. (2015) studied cold resistance of Olea europaea and found increased soluble sugar content in the detached leaves with the decrease of the stress temperature, but a larger increase in varieties with strong cold resistance. The study on vines also proved that the total soluble sugar content of the branches was significantly correlated with LT50 value (Ershadi et al., 2016; Zhao et al., 2020). In this study, the content of soluble sugar was highest in January, and was higher in BLF treated plants than in CK-treated plants in January 2021 and February 2022 (**Figure 7A**), with a significant negative correlation with EL-LT50 both years (**Figure 8**). The change in starch content shows the opposite trend (**Figures 7G, H**), and starch content in both years was significantly positively correlated with EL-LT50 (**Figure 8**), which may be due to the large amount of soluble sugar produced by the hydrolysis of starch. The concentrations of fructose, glucose, sucrose, raffinose, and stachyose in buds are strongly correlated with EL-LT50 (Grant and Dami, 2015), and sucrose is considered to be closely related to cold resistance (Guy et al., 1992). Sucrose and reducing sugar contents were also negatively correlated with EL-LT50 both years (**Figure 8**), and these results suggest that carbohydrates play an important role in the improvement of FT by BLF treatment. Sucrose also act as a signal molecule and can induce or inhibit gene expression to alter the physiological adaptability of plants (Koch, 1996; Smeekens and Rook, 1997). Future work should examine the content change and transformation mechanism of different soluble sugars during the wintering period of grapevines under BLF treatment.

### BLF treatment improved the freezing tolerance of grape cuttings by up-regulating the expression of CBFs

DREB is one of the main subfamilies of the AP2/ERF family, which is involved in the synergistic or antagonistic regulation of a variety of abiotic stresses, and plays a key role in the response of plants to various stresses such as cold, heat, drought, and high salinity (Licausi et al., 2013). The CBF/DREB1 transcription factors have been characterized as a regulatory hub in freezing tolerance in plant systems (Thomashow, 2010; Theocharis et al., 2012). Xiao et al., 2006; Xiao et al., 2008) reported that ABA and low temperature up-regulated the expression of CBF/DREB1 gene in grape leaves and seedlings. Rubio et al. (2018) found that the expression levels of VvCBFs genes were associated with the cold tolerance of grape dormant buds. In addition, heterologous overexpression of the VvCBF4 gene also reduced freezing-induced electrolyte leakage and improved freezing survival in non-cold-acclimated vines (Tillett et al., 2012). In this study, low temperature (LT) also up-regulated the expression of *CBF1* and *CBF3* genes in dormant cuttings, and due to the combined action of BLF and LT, the expression of VvCBF/DREB1 transcription factor was greatly increased, which reduced the electrolyte leakage caused by freezing in the branches. The accumulation of soluble sugars is a common biochemical process during cold acclimation of plants, but some studies have shown that overexpression of CBFs can also lead to changes in sugars. Gilmour et al. (2000) found that CBF3-overexpressing Arabidopsis plants had elevated levels of total soluble sugars, including sucrose, raffinose, glucose, and fructose. The increase of soluble sugar, reducing sugar and sucrose content in BLF-treated vines in this experiment may also be related to the up-regulation of CBFs. Ectopic expression of *VvICE1a* and *VvICE1b* isolated from ‘Muscat Hamburg’ grapevine activated the expression levels of *AtRD29A* and *AtCOR47* transcripts in transgenic Arabidopsis, enhancing tolerance to cold, drought, salinity, and cold-drought stress (Li et al., 2014). Dong et al. (2013) reported the inducer of CBF expression 1 (*ICE1*) from Vitis amurensis is strongly induced in leaves, roots, stems, and petioles by cold temperature and transgenic tobacco over-expressing VaICE1 has higher chilling tolerance and survival ability by improving the activities of superoxide dismutase, peroxidase, and catalase, as well as the chlorophyll yield. In this study, low temperature did not show a significant up-regulation of *ICE1*, and the relative expression of *ICE1* between BLF treatment and CK only showed a significant difference at 12 h of low temperature stress.

## Conclusion

Low temperature during overwintering resulted in the damage of grapevine manifested as the increase of O^2-^ and MDA, and the death of above-ground parts, branches and buds after overwintering. BLF treatment reduced mortality in all parts of the field vines after overwintering, and reduced the electrolyte leakage of branches in January. BLF treatment improved the antioxidant system by increasing POD and CAT activities, and decreased O^2-^ and MDA levels. BLF treatment increased the content of soluble protein and soluble carbohydrates, and alleviated the osmotic stress caused by low temperature and water loss during overwintering. BLF treatment up-regulated the expression levels of *CBF1* and *CBF3* in cuttings under low temperature stress, therefore improving freezing resistance. This study provide a theoretical basis for the study of winter protection measures for wine grapes in cold regions.

## Data availability statement

The original contributions presented in the study are included in the article/supplementary material. Further inquiries can be directed to the corresponding author.

## Author contributions

All authors contributed signifificantly to this manuscript. HL, HW, and XH conceived the project and designed the experiments. This article was primarily organized by XH, who participated in the entire process from experimental design to sample collection, indicators determination, data analysis, and article1 writing. FY, Y-HL, Z-LW, YW, XC, MH and DW made contributions to the determination of indicators, and T-TX made contributions to experimental treatment and sample collection. All authors have read and agreed to the published version of the manuscript.

## Funding

This research was supported by the National Key Research and Development Project (2019YFD1002500) and Key Research and Development Project of Shaanxi Province (2020ZDLNY07-08).

## Acknowledgments

The authors are grateful to the two engineers Huaitang Zheng and Xinyi Dong of the Great Winery for their help and support in the field trials.

## Conflict of interest

The authors declare that the research was conducted in the absence of any commercial or financial relationships that could be construed as a potential conflict of interest.

## Publisher’s note

All claims expressed in this article are solely those of the authors and do not necessarily represent those of their affiliated organizations, or those of the publisher, the editors and the reviewers. Any product that may be evaluated in this article, or claim that may be made by its manufacturer, is not guaranteed or endorsed by the publisher.

## References

[B1] Abeynayake S. W. Etzerodt T. P. Jonaviciene K. Byrne S. Asp T. Boelt B. (2015). Fructan metabolism and changes in fructan composition during cold acclimation in perennial ryegrass. Front. Plant Science. 6. doi: 10.3389/fpls.2015.00329 PMC4428078 26029229

[B2] Aebi H. (1984). Catalase *in vitro* . Methods Enzymol. 105, 121–126. doi: 10.1016/s0076-6879(84)05016-3 6727660

[B3] Anjum N. A. Ahmad I. Mohmood I. Pacheco M. Duarte A. C. Pereira E.. (2012). Modulation of glutathione and its related enzymes in plants’ responses to toxic metals and metalloids–a review. Environ. Exp. Botany 75, 307–324. doi: 10.1016/j.envexpbot.2011.07.002

[B4] Bates L. S. Waldren R. P. Teare I. D. (1973). Rapid determination of free proline for water-stress studies. Plant Soil. 39, 205–207. doi: 10.1007/BF00018060

[B5] Bhowmik P. K. Tamura K. I. Sanada Y. Tase K. Yamada T. (2006). Sucrose metabolism of perennial ryegrass in relation to cold acclimation. Z. Für Naturforschung C J. Biosciences. 61 (1-2), 99–104. doi: 10.1515/znc-2006-1-218 16610225

[B6] Bourne T. F. Moore J. N. (1991). Cold hardiness in grape cultivar development. Fruit Varieties J. 45, 26–28.

[B7] Bradford M. M. (1976). A rapid and sensitive method for the quantitation of microgram quantities of protein utilizing the principle of protein-dye binding. Analytical Biochem. 72, 248–254. doi: 10.1006/abio.1976.9999 942051

[B8] Chai C. J. Wang M. Z. (1996). Effects of cold-resistant rootstocks on moisture changes and safety of overwintering grape vines in open fields. Tianjin Agric. Sci. 2, 20–21.

[B9] Chen T. H. H. Burke M. J. Gusta L. V. (1995). Freezing tolerance in plants: an overview (St Paul, Minnesota: APS Press).

[B10] Chen L. Y. Z. Xu S. Zhang Z. Xu Y. Zhang J. (2018). OsMADS57 together with OsTB1 coordinates transcription of its target *OsWRKY94* and *D14* to switch its organogenesis to defense for cold adaptation in rice. New Phytol. 218, 219–231. doi: 10.1111/nph.14977 29364524 PMC5873253

[B11] Chen B. H. Zhang B. Mao J. Hao Y. Yang R. Cai X. H.. (2014). The relationship between the changing of water content and the cold resistance of grape branches. Plant Physiol. J. 50, 535–541. doi: 10.13592/j.cnki.ppj.2013.0441

[B12] Chinnusamy V. Ohta M. Kanrar S. Lee B. Hong X. Agarwal M.. (2003). ICE1: a regulator of cold-induced transcriptome and freezing tolerance in arabidopsis. Genes Dev. 17, 1043–1054. doi: 10.1101/gad.1077503 12672693 PMC196034

[B13] Chinnusamy V. Zhu J. Zhu J. K. (2007). Cold stress regulation of gene expression in plants. Trends Plant Sci. 12 10, 444–451. doi: 10.1016/j.tplants.2007.07.002 17855156

[B14] Dong C. Zhang Z. Ren J. P. Qin Y. Huang J. F. Wang Y.. (2013). Stress-responsive gene ICE1 from *Vitis amurensis* increases cold tolerance in tobacco. Plant Physiol. Biochem. 71, 212–217. doi: 10.1016/j.plaphy.2013.07.012 23968929

[B15] Elstner E. F. Heupel A. (1976). Inhibition of nitrite formation from hydroxylammoniumchloride: a simple assay for superoxide dismutase. Analytical Biochem. 70 2, 616–620. doi: 10.1016/0003-2697(76)90488-7 817618

[B16] Ershadi A. Karimi R. Mahdei K. N. (2016). Freezing tolerance and its relationship with soluble carbohydrates, proline and water content in 12 grapevine cultivars. Acta Physiologiae Plantarum. 38, 2. doi: 10.1007/s11738-015-2021-6

[B17] Fuller M. P. Grice P. L. (1998). A chamber for the simulation of radiation freezing of plants. Ann. Appl. Biol. 133, 111–121. doi: 10.1111/j.1744-7348.1998.tb05807.x

[B18] Fuller M. P. Hamed F. Wisniewski M. Glenn D. M. (2003). Protection of plants from frost using hydrophobic particle film and acrylic polymer. Ann. Appl. Biol. 143, 93–98. doi: 10.1111/j.1744-7348.2003.tb00273.x

[B19] Fuller M. P. Wisniewski M. (1998). The use of infrared thermal imaging in the study of ice nucleation and freezing of plants. J. Thermal Biol. 23 (2), 81–89. doi: 10.1016/S0306-4565(98)00013-8

[B20] Gao J. F. (2006). Experimental guidance for plant physiology (Beijing, China: Higher Education Press).

[B21] Giannopolitis C. N. Ries S. K. (1977). Superoxide dismutases: I. occurrence in higher plants. Plant Physiol. 59, 309–314. doi: 10.1104/pp.59.2.309 16659839 PMC542387

[B22] Gilmour S. J. Audrey M. Maite P. Everard J. D. Thomashow M. F. (2000). Overexpression of the arabidopsis CBF3 transcriptional activator mimics multiple biochemical changes associated with cold acclimation. Plant Physiol. 124 (4), 1854–1865. doi: 10.1104/pp.124.4.1854 11115899 PMC59880

[B23] Grant T. Dami I. E. (2015). Physiological and biochemical seasonal changes in vitis genotypes with contrasting freezing tolerance. Am. J. Enology Viticulture. 66, 195 -203. doi: 10.5344/ajev.2014.14101

[B24] Grossnickle S. C. South D. B. (2014). Fall acclimation and the lift/store pathway: effect on reforestation. Open For. Sci. J. 714 1, 1–20. doi: 10.2174/1874398601407010001

[B25] Guy C. L. (1990). Cold acclimation and freezing stress tolerance: role of protein metabolism. Annu. Rev. Plant Physiol. Plant Mol. Biol. 41, 187–223. doi: 10.1146/annurev.pp.41.060190.001155

[B26] Guy C. L. Huber J. L. Huber S. (1992). Sucrose phosphate synthase and sucrose accumulation at low temperature. Plant Physiol. 100, 502–508. doi: 10.1104/pp.100.1.502 16652990 PMC1075578

[B27] Haghi H. Rabiei V. Ershadi A. Razavi F. (2019). Effects of late season foliar application of calcium chloride on cold hardiness in grapevines (*Vitis vinifera* ‘Thompson seedless’). Horticulture J. 88, 347–353. doi: 10.2503/hortj.UTD-056

[B28] Han X. Xue T. T. Liu X. Wang Z. L. Zhang L. Wang Y.. (2021). A sustainable viticulture method adapted to the cold climate zone in China. Horticulturae 7, 150. doi: 10.3390/horticulturae7060150

[B29] Hasanuzzaman M. Bhuyan M. H. M. B. Zulfiqar F. Raza A. Al Mahmud J. Fujita M.. (2020). Reactive oxygen species and antioxidant defense in plants under abiotic stress: revisiting the crucial role of a universal defense regulator. Antioxidants. 9, 681. doi: 10.3390/antiox9080681 PMC7465626 32751256

[B30] Hashempour A. Ghasemnezhad M. Ghazvini R. F. Sohani M. M. (2014). Olive (*Olea europaea* l.) freezing tolerance related to antioxidant enzymes activity during cold acclimation and non acclimation. Acta Physiologiae Plantarum. 36, 3231–3241. doi: 10.1007/s11738-014-1689-3

[B31] Hodges D. M. DeLong J. M. Forney C. F. Prange R. K. (1999). Improving the thiobarbituric acid-reactive-substances assay for estimating lipid peroxidation in plant tissues containing anthocyanin and other interfering compounds. Planta. 207, 604–611. doi: 10.1007/s004250050524 28456836

[B32] Jolivet Y. Dubois J. M. M. (2000). Evaluation of hilling efficiency as a method of protection of vine against winter cold in québec. J. Int. Des. Sci. la Vigne du vin. 3, 83–92. doi: 10.20870/oeno-one.2000.34.3.1001

[B33] Kaya O. (2020a). Defoliation alleviates cold-induced oxidative damage in dormant buds of grapevine by up-regulating soluble carbohydrates and decreasing ROS. Acta Physiologiae Plantarum 42 (7), 1–10. doi: 10.1007/s11738-020-03093-1

[B34] Kaya O. (2020b). Bud death and its relationship with lateral shoot, water content and soluble carbohydrates in four grapevine cultivars following winter cold. Erwerbs-Obstbau 62 (1), 43–50. doi: 10.1007/s10341-020-00495-w

[B35] Kaya Ö. Köse C. (2017). Determination of resistance to low temperatures of winter buds on lateral shoot present in karaerik (Vitis vinifera l.) grape cultivar. Acta Physiologiae Plantarum 39 (9), 1–9. doi: 10.1007/s11738-017-2513-7

[B36] Kaya O. Kose C. (2020). How pretest temperatures change the cold hardiness of grapevine (Vitis vinifera l. cv. karaerik) dormant buds? Int. J. Fruit Sci. 20 (Suppl 3), S1470–S1482. doi: 10.1080/15538362.2020.1804516

[B37] Kaya O. Kose C. Donderalp V. Gecim T. Taskın S. (2020). Last updates on cell death point, bud death time and exothermic characteristics of flower buds for deciduous fruit species by using differential thermal analysis. Scientia Hortic. 270, 109403. doi: 10.1016/j.scienta.2020

[B38] Kaya O. Kose C. Esitken A. Gecim T. Donderalp V. Taskin S.. (2021). Frost tolerance in apricot (*Prunus armeniaca* l.) receptacle and pistil organs: how is the relationship among amino acids, minerals, and cell death points? Int. J. Biometeorology 65 (12), 2157–2170. doi: 10.1007/s00484-021-02178-x 34324064

[B39] Kaya O. Kose C. Esıtken A. Turan M. Utku O. (2021). Can organic acid and sugar compositions be used to predict cell death point limits? receptacle and pistil organs of apricot (*Prunus armeniaca* l.). Rendiconti Lincei. Sci. Fisiche e Naturali 32 (3), 493–509. doi: 10.1007/s12210-021-01007-y

[B40] Khanizadeh S. Rekika D. Levasseur A. Groleau Y. Fisher H. (2005). The effects of different cultural and environmental factors on grapevine growth, winter hardiness and performance, in three locations, in Canada. Small Fruits Rev. 4, 3–28. doi: 10.1300/J301v04n03_02

[B41] Kim Y. S. Lee M. Lee J. H. Lee H. J. Park C. M. (2015). The unified ICE-CBF pathway provides a transcriptional feedback control of freezing tolerance during cold acclimation in arabidopsis. Plant Mol. Biol. 89 (1-2), 187–201. doi: 10.1007/s11103-015-0365-3 26311645

[B42] Koch K. E. (1996). Carbohydrate-modulated gene expression in plants. Annu. Rev. Plant Physiol. Plant Mol. Biol. 47, 509–540. doi: 10.1146/annurev.arplant.47.1.509 15012299

[B43] Lan Y. C. Shen L. X. Li R. F. (2013). Effects of different film mulching on soil temperature and moisture. Chin. Agric. Sci. Bulletin. 29, 120–126.

[B44] Lee S. P. Chen T. H. H. (1992). Molecular biology of plant cold hardiness development. In (eds) Adv. Plant Cold hardiness. CRC Press Boca Raton pp, 1–30.

[B45] Li H. (2015). Crawled cordon training: A new grapevine shaping and pruning system for the soil-bury over-wintering zone in China (Yangling, Shaanxi: NWSUAF Press).

[B46] Licausi F. Ohme-Takagi M. Perata P. (2013). APETALA2/Ethylene responsive factor (AP2/ERF) transcription factors: mediators of stress responses and developmental programs. N Phytologist. 199, 639–649. doi: 10.1111/nph.12291 24010138

[B47] Li C. Y. Chen S. S. Xu W. Li D. S. Gu X. Zhu X. K.. (2011). Effect of low temperature at seedling stage on antioxidation enzymes and cytoplasmic osmoticum of leaves in wheat cultivar yangmai 16. Acta Agronomica Sinica. 37, 2293–2298. doi: 10.3724/SP.J.1006.2011.02293

[B48] Ling F. Jiao J. Li C. Z. Jin Q. X. Zhao M. L. (2015). Physiological reaponse and comprehensive evaluation of cold resistance under cold stress for different varieties of olea europaea. Acta Botanica Boreali Occidentalia Sinica. 35, 0508–0515. doi: 10.7606/j.issn.1000-4025.2015.03.0508

[B49] Liu Q. Kasuga M. Sakuma Y. Abe H. Miura S. Yamaguchi –Shinozaki K.. (1998). Two transcription factors, DREB1 and DREB2, with an EREBP/AP2 DNA binding domain separate two cellular signal transduction pathways in drought- and low-temperature-responsive gene expression, respectively, in arabidopsis. Plant Cell. 10 (8), 1391–1406. doi: 10.1105/tpc.10.8.1391 9707537 PMC144379

[B50] Liu L. Li S. X. Guo J. H. Li N. Jiang M. Li X. N. (2022). Low temperature tolerance is depressed in wild-type and abscisic acid-deficient mutant barley grown in cd-contaminated soil. J. Hazardous Materials 430, 128489. doi: 10.1016/j.jhazmat.2022.128489 35739670

[B51] Liu J. Wang X. W. Wei X. P. Lu R. Q. Gao Z. Q. (2004). Achievement and prospect of world cold-resistance grape breeding. J. Fruit Sci. 21, 461–466. doi: 10.1007/BF02873091

[B52] Livak K. J. Schmittgen T. D. (2001). Analysis of relative gene expression data using real-time quantitative PCR and the 2^-CT^ method. Methods. 25, 402–408. doi: 10.1006/meth.2001.1262 11846609

[B53] Li J. T. Wang L. N. Zhu W. Wang N. A. Xin H. P. Li S. H. (2014). Characterization of two VvICE1 genes isolated from ‘Muscat hamburg’ grapevine and their effect on the tolerance to abiotic stresses. Scientia Hortic. 165, 266–273. doi: 10.1016/j.scienta.2013.11.002

[B54] Lu M. (2006). Effect of humic acid as special organic fertilizer for eucalyptus on cold resistance and growth stimulation of eucalyptus dunnii. J. Zhejiang Forestry College. 23, 501–506.

[B55] Lv D. K. Xi B. Yong L. Ding X. D. Ge Y. Cai H.. (2010). Profiling of cold-stress-responsive miRNAs in rice by microarrays. Gene. 459, 39–47. doi: 10.1016/j.gene.2010.03.011 20350593

[B56] Lyons J. M. (1973). Chilling injury in plants. Annu. Rev. Plant Physiol. 24, 445–466. doi: 10.1146/annurev.pp.24.060173.002305

[B57] Meiering A. G. Paroschy J. H. Peterson R. L. Hostetter G. Neff A. (1980). Mechanical freezing injury in grapevine trunks. Am. J. Enology Viticulture. 31, 81–89.

[B58] Pei D. L. (2012). The progress of GSTs in plant defense. Chin. Agric. Sci. Bull. 28, 185–188.

[B59] Pierquet P. Stushnoff C. Burke M. J. (1977). Low temperature exotherms in stem and bud tissues of vitis riparia michx. J. Am. Soc. Hortic. Sci. Am. Soc. Hortic. Sci. 102, 54–55. doi: 10.21273/JASHS.102.1.54

[B60] Puhakainen T. Li C. Y. Boije-Malm M. Heino P. Palva E. T. (2004). Short-day potentiation of low temperature-induced gene expression of a c-Repeat-Binding factor-controlled gene during cold acclimation in silver birch. Plant Physiol. 136 4, 4299–4307. doi: 10.1104/pp.104.047258 15563624 PMC535859

[B61] Rao M. Paliyath G. Ormrod D. P. (1996). Ultraviolet-b- and ozone-induced biochemical changes in antioxidant enzymes of arabidopsis thaliana. Plant Physiol. 110, 125–136. doi: 10.1104/pp.110.1.125 8587977 PMC157701

[B62] Rende M. Kose C. Kaya O. (2018). An assessment of the relation between cold-hardiness and biochemical contents of winter buds of grapevine cv.’Karaerik’in acclimation-hardening-deacclimation phases. Mitt. Klosterneuburg Rebe und Wein Obstbau und Früchteverwertung 68 (2), 67–81.

[B63] Ren J. Huang Z. L. Zeng L. X. Shi Z. (2013). A review of physiological reaction mechanism of plants exposed to low temperature stress. World Forestry Res. 26, 15–20. doi: 10.13348/j.cnki.sjlyyj.2013.06.011

[B64] Ritonga F. N. Chen S. (2020). Physiological and molecular mechanism involved in cold stress tolerance in plants. Plants. 9 (5), 560. doi: 10.3390/plants9050560 PMC7284489 32353940

[B65] Rubio S. Noriega X. Pérez F. J. (2018). Abscisic acid (ABA) and low temperatures synergistically increase the expression of CBF/DREB1 transcription factors and cold-hardiness in grapevine dormant buds. Ann. Botany. 123, 681–689. doi: 10.1093/aob/mcy201 PMC6417478 30418484

[B66] Salzman R. A. Bressan R. A. Hasegawa P. M. Ashworth E. N. Bordelon B. P. (1996). Programmed accumulation of LEA-like proteins during desiccation and cold acclimation of overwintering grape buds. Plant Cell Environment. 19, 713–720. doi: 10.1111/j.1365-3040.1996.tb00406.x

[B67] Schnabel B. J. Wample R. L. (1987). Dormancy and cold hardiness in vitis vinifera l. cv. white Riesling as influenced by photoperiod and temperature. Am. J. enology viticulture. 38, 265–272.

[B68] Shi Y. T. Ding Y. L. Yang S. H. (2018). Molecular regulation of CBF signaling in cold acclimation. Trends Plant Sci. 23 (7), 623–637. doi: 10.1016/j.tplants.2018.04.002 29735429

[B69] Siddiqua M. Nassuth A. (2011). Vitis CBF1 and vitis CBF4 differ in their effect on arabidopsis abiotic stress tolerance, development and gene expressionpce. Plant Cell Environment. 34, 1345–1359. doi: 10.1111/j.1365-3040.2011.02334.x 21486303

[B70] Smeekens S. Rook F. (1997). Sugar sensing and sugar-mediated signal transduction in plants. Plant Physiol. 115 >1, 7–13. doi: 10.1104/pp.115.1.7 12223788 PMC158454

[B71] Snyder R. L. Melo-Abreu J. P. D. Matulich S. (2005). Frost protection: fundamentals, practice and economics (Rome, Italy: Food and Agriculture Organization of the United Nations).

[B72] Soloklui A. A. G. Ershadi A. Fallahi E. (2012). Evaluation of cold hardiness in seven Iranian commercial pomegranate (Punica granatum l.) cultivars. Hortscience. 47, 1821–1825. doi: 10.21273/HORTSCI.47.12.1821

[B73] Stockinger E. J. Gi lmour S. J. Thomashow M. F. (1997). Arabidopsis thaliana *CBF1* encodes an AP2 domain-containing transcriptional activator that binds to the c-repeat/DRE, a cis-acting DNA regulatory element that stimulates transcription in response to low temperature and water deficit. Proc. Natl. Acad. Sci. U. S. A. 94 (3), 1035–1040. doi: 10.1073/pnas.94.3.1035 9023378 PMC19635

[B74] Theocharis A. Clement C. Barka E. A. (2012). Physiological and molecular changes in plants grown at low temperatures. Planta. 235 6, 1091–1105. doi: 10.1007/s00425-012-1641-y 22526498

[B75] Thomashow M. F. (1999). PLANT COLD ACCLIMATION: Freezing tolerance genes and regulatory mechanisms. Annu. Rev. Plant Physiol. Plant Mol. Biol. 50, 571–599. doi: 10.1146/annurev.arplant.50.1.571 15012220

[B76] Thomashow M. F. (2010). Molecular basis of plant cold acclimation: insights gained from studying the CBF cold response pathway. Plant Physiol. 154, 571–577. doi: 10.1104/pp.110.161794 20921187 PMC2948992

[B77] Tillett R. L. Wheatley M. D. Tattersall E. A. Schlauch K. A. Cramer G. R. Cushman J. C. (2012). The vitis vinifera c-repeat binding protein 4 (*VvCBF4*) transcriptional factor enhances freezing tolerance in wine grape. Plant Biotechnol. J. 10, 105–124. doi: 10.1111/j.1467-7652.2011.00648.x 21914113 PMC4357522

[B78] Uemura M. Steponkus P. L. (1994). A contrast of the plasma membrane lipid composition of oat and rye leaves in relation to freezing tolerance. Plant Physiol. 104, 479–496. doi: 10.1104/pp.104.2.479 12232097 PMC159222

[B79] Vadim D. Darya S. Medvedev S. S. Pozhvanov G. A. Anatoliy S. Vladimir Y. (2014). Stress-induced electrolyte leakage: the role of k+-permeable channels and involvement in programmed cell death and metabolic adjustment. J. Exp. Botany. 65, 1259–1270. doi: 10.1093/jxb/eru004 24520019

[B80] Wang S. (2015). Effect of shoots windbreak on vineyard ecotope in the soil-buried cold-proof period (Yangling, China: Northwest A&F University).

[B81] Wang Z. S. Li S. X. Jian S. L. Ye F. Wang T. Y. Gong L.. (2022). Low temperature tolerance is impaired by polystyrene nanoplastics accumulated in cells of barley (*Hordeum vulgare* l.) plants. J. Hazardous Materials 426, 127826. doi: 10.1016/j.jhazmat.2021.127826 34823951

[B82] Wang H. T. Ma X. F. Zhang J. Z. (2010). The application of humic acid in soil and fertilizer. Heilongjiang Sci. 1, 59–62.

[B83] Wang X. Xu G. B. Ren Z. G. Zhang Z. J. Jian Y. F. Zhang Y. M. (2007a). Effects of environment-friendly degradable films on corn growth and soil environment. Chin. J. Eco-Agriculture. 15, 78–81.

[B84] Wang W. J. Zhang Y. H. Niu J. F. Wang Z. P. (2007b). Study on cold tolerance of table grape cultivars by measuring the conductivity. J. Fruit Science. 24, 34–37. doi: 10.13925/j.cnki.gsxb.2007.01.008

[B85] Weiser C. J. (1970). Cold resistance and injury in woody plants. Science. 169, 1269–1278. doi: 10.1126/science.169.3952.1269 17772511

[B86] Wisniewski M. Bassett C. Gusta L. V. (2003). An overview of cold hardiness in woody plants: Seeing the forest through the trees. Hortscience. 38, 952–959. doi: 10.21273/HORTSCI.38.5.952

[B87] Wisniewski M. Glenn D. M. Fuller M. P. (2002). Use of a hydrophobic particle film as a barrier to extrinsic ice nucleation in tomato plants. journal of the American society for. Hortic. Science. 127, 358–364. doi: 10.21273/JASHS.127.3.358

[B88] Xiao H. Siddiqua M. Braybrook S. Nassuth A. (2006). Three grape CBF / DREB1 genes respond to low temperature, drought and abscisic acid. Plant Cell Environment. 29, 1410–1421. doi: 10.1111/j.1365-3040.2006.01524.x 17080962

[B89] Xiao H. Tattersall E. Siddiqua M. Cramer G. Nassuth A. (2008). *CBF4* is a unique member of the CBF transcription factor family of vitis vinifera and vitis riparia. Plant Cell Environment. 31 1, 1–10. doi: 10.1111/j.1365-3040.2007.01741.x 17971068

[B90] Xue T. T. (2019a). Research of free-buried mode based on biodegradable liquid film in grapevine soil-burial areas (Yangling, China: Northwest A&F University).

[B91] Xue T. T. Han X. Zhang H. J. Wang Y. Wang H. Li H. (2019b). Effects of a biodegradable liquid film on winter chill protection of winegrape cultivars. Scientia Hortic. 246, 398–406. doi: 10.1016/j.scienta.2018.11.013

[B92] Yamaguchi-Shinozaki K. Shinozaki K. (1994). A novel cis-acting element in an arabidopsis gene is involved in responsiveness to drought, low-temperature, or high-salt stress. Plant Cell. 6 (2), 251–264. doi: 10.1105/tpc.6.2.251 8148648 PMC160431

[B93] Yang Y. Jia Z. K. Chen F. J. Sang Z. Y. Ma L. Y. (2015). Comparative analysis of natural cold acclimation and deacclimation of two magnolia species with different winter hardiness. Acta Physiologiae Plantarum. 37, 129. doi: 10.1007/s11738-015-1883-y

[B94] Yemm E. W. Willis A. J. (1954). The estimation of carbohydrates in plant extracts by anthrone. Biochem. J. 57, 508–514. doi: 10.1042/bj0570508 13181867 PMC1269789

[B95] Zhang G. Ryyppo A. Repo T. (2002). The electrical impedance spectroscopy of scots pine needles during cold acclimation. Physiologia Plantarum 115, 385–392. doi: 10.1034/j.1399-3054.2002.1150308.x 12081531

[B96] Zhang J. Wu X. Niu R. Liu Y. Liu N. Xu W.. (2012). Cold-resistance evaluation in 25 wild grape species. Vitis. 51 4, 153–160.

[B97] Zhao Y. Wang Z. X. Yang Y. M. Liu H. S. Ai J. (2020). Analysis of the cold tolerance and physiological response differences of amur grape (Vitis amurensis) germplasms during overwintering. Scientia Horticulturae. 259, 1–9. doi: 10.1016/j.scienta.2019.108760

